# Integrated Transcriptomic Analyses of Liver and Mammary Gland Tissues Reveals the Regulatory Mechanism Underlying Dairy Goats at Late Lactation When Feeding Rumen-Protected Lysine

**DOI:** 10.3390/ijms252111376

**Published:** 2024-10-23

**Authors:** Wenting Dai, Bingqing Han, Yalu Sun, Pengfei Hou, Chong Wang, Weini Li, Hongyun Liu

**Affiliations:** 1 College of Animal Sciences, Zhejiang University, Hangzhou 310058, China; daiwenting@zjut.edu.cn (W.D.); 21917010@zju.edu.cn (B.H.); sunyalu543@126.com (Y.S.); 21817012@zju.edu.cn (P.H.); 2 College of Pharmaceutical Science, Zhejiang University of Technology, Hangzhou 310032, China; 3 College of Animal Sciences and Technology, Zhejiang A & F University, Lin’an 311300, China; wangcong992@163.com; 4 Department of Biomedical Science, Cedars-Sinai Medical Center, Cedars-Sinai Cancer Institute, Los Angeles, CA 90067, USA; liweini19880910@gmail.com

**Keywords:** transcriptomic analysis, rumen-protected lysine, liver, mammary gland, late lactation, dairy goats

## Abstract

Although low-protein diets can improve the nitrogen utilization efficiency and alleviate economic pressures in ruminants, they may also negatively impact dairy performance. Rumen-protected lysine (RPL) supplementation can improve the health status and growth performance of ruminants without compromising nitrogen utilization efficiency and feed intake. In this study, a total of thirty-three multiparous dairy goats in the late-lactation period were randomly divided into three groups that were separately fed the control diet (namely the protein-adequacy group), the low-protein diet (namely the protein-deficient group), and the RPL-supplemented protein-deficient diet (namely RPL-supplementation group) for five weeks. Here, we investigated the molecular mechanisms regarding how low-protein diets with RPL supplementation compromise lactation phenotypes in dairy goats through cross-tissue transcriptomic analyses. Dietary protein deficiency caused an imbalance in amino acid (AA) intake, disrupted hepatic function, and impaired milk synthesis. Transcriptomic analyses further showed that RPL supplementation exhibited some beneficial effects, like mitigating abnormal lipid and energy metabolism in the liver, elevating hepatic resistance to oxidative stress, improving the mammary absorption of AAs, as well as activating mammary lipid and protein anabolism primarily through peroxisome proliferator-activated receptor (PPAR) and janus kinase-signal transducer (JAK)—signal transducer and activator of transcription (STAT) signaling, respectively. RPL supplementation of a low-protein diet contributes to maintaining late lactation in dairy goats primarily through mitigating hepatic energy disturbances and activating both lipid and protein metabolism in the mammary glands. Since RPL supplementation initiated a series of comprised events on mammary protein and lipid metabolism as well as the hepatic function and energy generation in dairy goats under protein deficiency during late lactation, these findings thus provide some insights into how RPL supplementation helps maintain milk production and health in dairy mammals especially at late lactation.

## 1. Introduction

As the global demand for dairy products continues to rise, the scarcity of available feed resources requires an enhancement in feed utilization efficiency. Previous studies have demonstrated that diets with a reduced protein content can improve nitrogen (N) utilization and decrease feed costs in ruminants. However, these advantages are accompanied by a decline in both growth and lactation performances [1]. For instance, dairy cows fed with low-protein diets exhibited a marked decrease in milk yield during the mid-lactation and post-lactation periods [2]. Furthermore, the reduction in dietary protein levels led to an amino acid (AA) deficiency, which in turn compromised production performance [3].

Supplementation with rumen-protected lysine (RPL) has been demonstrated to enhance the growth performance and overall health status of ruminants. For instance, the addition of RPL during the prepartum and postpartum periods has been found to improve uterine health and elevate the number of mammary epithelial cells, which in turn led to an increase in dry matter intake (DMI), energy-corrected milk (ECM), and milk yield [4,5]. Moreover, RPL supplementation of low-protein diets improved intestinal mucosal integrity, host immune function, and gut microbiota composition in growing lambs [5]. Also, maintaining lactation performance while increasing N utilization in ruminants remains a significant research focus. These studies are limited to the productive studies regarding how RPL exerts beneficial effects in dairy mammals. With the development of omics techniques, the related studies have been utilized to evaluate the underlying mechanisms for more in-depth and systematic exploration in a single organ or even across multiple organs.

In this study, we investigated the impact of RPL on the lactation performance of late-lactating dairy goats and its underlying mechanism at the cross-tissue levels. Through individual and integrated transcriptome analyses of liver and mammary gland tissues, we explored the effects of lysine supplementation of low-protein diets on lactation performance in dairy goats. Importantly, we revealed that RPL supplementation maintains lactation in dairy goats by compromising the imbalance in AA intake, mitigating hepatic abnormal function, and activating mammary lipid and protein anabolism. Overall, our study elucidates the regulatory mechanisms by which RPL supplementation under protein deficiency affects the late lactation of dairy goats. This study offers valuable insights for optimizing dairy goat nutrition and management practices.

## 2. Results

### 2.1. Lactation Performance

Among the three groups, no significant differences were observed in DMI and several parameters, including milk yield, milk composition (g/d), 4% fat-corrected milk (FCM), and ECM, as well as the composition of milk such as milk protein, milk fat, and lactose (*p* > 0.05, Table 1). Despite this, we surely observed a significantly higher concentration of milk protein under the RPL-supplemented metabolizable protein (MP)-deficient diet (*p* = 0.04), namely the DL group. Furthermore, the supplementation of a low-protein diet with RPL reduced the milk urea nitrogen (MUN) level (*p* = 0.04), while it elevated the N conversion, compared to the protein-deficient (D) group.

### 2.2. AA Profiles in Plasma and Milk

In this study, we determined the effects of RPL supplementation on the AA profiles in both plasma and milk in dairy goats at late lactation. Firstly, we observed no significant differences in the concentrations of free AA from plasma among the three groups (*p* > 0.05, Table S2). By contrast, in milk, compared to the protein-adequacy (C) group, protein deficiency in the diet significantly elevated the levels of several essential AAs (arginine, histidine, leucine, and methionine), non-essential AAs (cysteine, glutamate, glycine, proline, and tyrosine), and branch-chain AAs (BCAAs) within the D group (*p* < 0.01, Table S3). Intriguingly, the RPL-supplemented protein-deficient diet significantly restored these AA levels in the D group, such as histidine, leucine, methionine, glutamate, and proline, to a similar level with those in the protein-adequacy (C) group (*p* < 0.01, Table S3).

### 2.3. Physiological and Biochemical Parameters in Plasma Associated with Protein Metabolism, Energy Metabolism, and Hepatic Metabolism

In the plasma, we detected no significant changes in most biochemical parameters involved in protein metabolism (including total protein, albumin (A), globulin (G), creatinine, and β-hydroxybutyric acid (B-HB)), energy metabolism (including glucose, triglyceride, and cholesterol), and hepatic metabolism (alanine aminotransferase (ALT), aspartate aminotransferase (AST), and alkaline phosphatase (ALP)) (Table S4). Notably, blood urea nitrogen (BUN) was significantly reduced in the protein-deficient (D) and RPL-supplementation (DL) groups, compared to the protein-adequacy (C) group (*p* = 0.04, Table S4). Importantly, the level of non-esterified fatty acids (NEFA), a crucial energy substrate, was significantly elevated when RPL was added to the protein deficiency diet (namely the DL group) (*p* < 0.01, Table S4). As an important marker of hepatic disease, AST was higher under MP deficiency compared to protein adequacy (in the D vs. C groups); on the contrary, this enzyme reduced after RPL was added to the MP deficiency diet (in the DL vs. D groups) (*p* = 0.02, Table S4).

### 2.4. Effects of RPL Supplementation on the Hepatic Transcriptomic Profile

**Figure 1:**
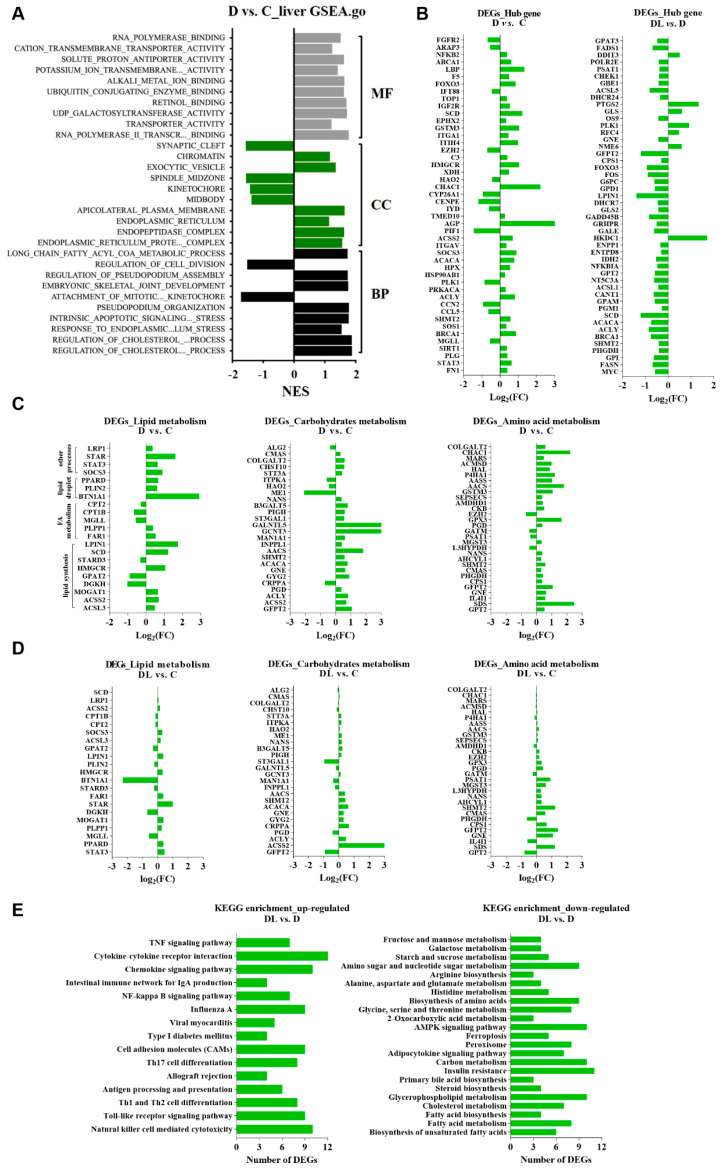
Effects of supplying lysine on hepatic transcriptome of dairy goats during late lactation. (**A**) GSEA analysis of DEGs from liver in D vs. C groups. MF in grey represents molecular function; CC in green represents cell component; BP in black represents biological process. (**B**) Hub genes from liver in D vs. C and DL vs. D groups. DEGs from liver in D vs. C (**C**) and DL vs. C (**D**) groups are involved in the lipid metabolism, carbohydrate metabolism, and amino acid metabolism. (**E**) KEGG analysis of the up-regulated and down-regulated DEGs from liver in DL vs. D groups. “C” represents the control group, namely protein-adequacy group; “D” represents the protein-deficient group; and “DL” represents the rumen-protected lysine (RPL)-supplemented protein-deficient group.

As depicted in Figure 1A, we firstly conducted a Gene Set Enrichment Analysis (GSEA) of all the differentially expressed genes (DEGs), and identified numerous hub genes related to nutrient metabolism in the livers both from the D vs. C (namely protein deficiency vs. protein adequacy) and DL vs. D (RPL supplementation vs. protein deficiency) groups (Figure 1B). Among them, hub genes from the D vs. C groups (namely protein deficiency vs. protein adequacy) were associated with “fatty acid metabolism”, “fatty acid biosynthesis”, and “glycerophospholipid metabolism”. Compared to the control (C) group, the abnormal expression of most genes was rescued after RPL supplementation. Moreover, these hub genes were primarily involved in “lipid metabolism”, “amino acid metabolism”, and “protein modification processes” in both the D vs. C (protein deficiency vs. protein adequacy, Figure 1C) and DL vs. C (RPL supplementation vs. protein adequacy, Figure 1D) groups. Notably, under protein deficiency, three DEGs, acetyl-CoA carboxylase alpha (*ACACA*), stearoyl-CoA desaturase (*SCD*), and xanthine dehydrogenase (*XDH*) were significantly enriched in “fatty acid biosynthesis-related processes”. Meanwhile, ATP citrate lyase (*ACLY*) and malic enzyme 1 (ME1) were significantly enriched in “carbon metabolism”. Furthermore, we next conducted a KEGG enrichment analysis of all the DEGs among different groups. In the up-regulated genes from the DL vs. D group (RPL supplementation vs. protein deficiency), 240 KEGG pathways were enriched in total; 15 of which were significant (Figure 1E); In contrast, a total of 287 KEGG pathways were enriched from the down-regulated genes, among which 23 pathways were significantly different (Figure 1E).

### 2.5. Effects of RPL Supplementation on the Mammary Gland Transcriptomic Profile

**Figure 2:**
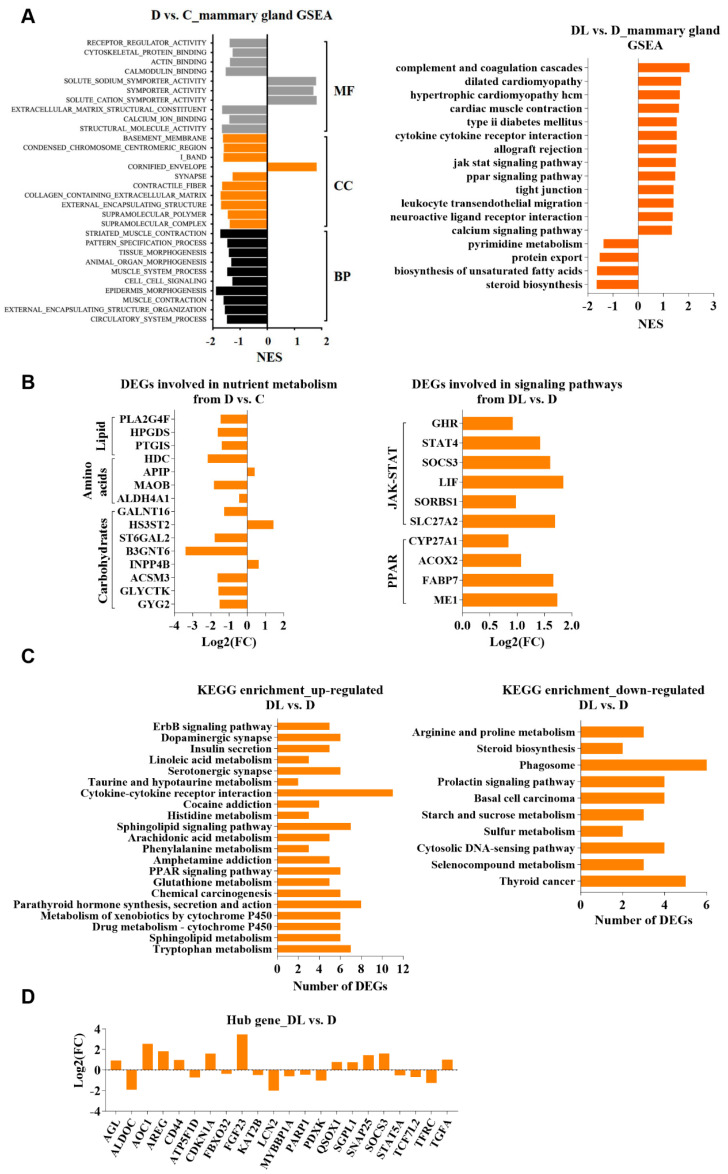
Effects of lysine supplementation on mammary gland transcriptome of dairy goats at late lactation. (**A**) GSEA analysis of DEGs from mammary glands in D vs. C and DL vs. D groups. (**B**) DEGs from mammary glands in D vs. C and DL vs. D groups are involved in the nutrient metabolism and signaling pathway. (**C**) KEGG analysis of the up-regulated and down-regulated DEGs from mammary glands in DL vs. D groups. (**D**) Hub genes from mammary glands in DL vs. D groups. “C” represents the control group, namely protein-adequacy group; “D” represents the protein-deficient group; and “DL” represents the rumen-protected-lysine-supplemented protein-deficient group.

Similarly, a GSEA enrichment analysis of mammary DEGs was conducted, and we detected numerous hub genes among the DEGs from mammary tissues in the D vs. C (protein deficiency vs. protein adequacy) groups and DL vs. D (RPL supplementation vs. protein deficiency) groups (Figure 2A). Remarkably, these genes were predominantly involved in lipid metabolism, carbon metabolism, and nitrogen metabolism in the mammary gland from the D vs. C (protein deficiency vs. protein adequacy) groups and two signaling pathways, Janus tyrosine kinase (JAK2)─Signal transducer and activator of transcription (STAT5) and peroxisome-proliferator-activated receptor (PPAR), from the DL vs. D (RPL supplementation vs. protein deficiency) groups (Figure 2B). Moreover, the GSEA analysis highlighted the significant enrichment of the JAK–STAT signaling pathway, including several key genes such as *STAT4*, suppressor of cytokine signaling 3 (*SOCS3*), AKT serine/threonine kinase 3 (*AKT3*), *JAK3*, and growth hormone receptor (*GHR*). Also, a couple of DEGs involved in the regulation of the PPAR signaling pathway, such as fatty acid binding protein 7 (*FABP7*), *ME1*, and acyl-CoA oxidase 2 (*ACOX2*), were significantly enriched (Figure 2B). Moreover, 230 KEGG pathways were totally enriched, with 21 being significantly different in the DL vs. D groups (RPL supplementation vs. protein deficiency, Figure 2C). However, 113 down-regulated genes from the DL vs. D groups were enriched in 219 KEGG pathways, among which only 10 pathways were significantly affected, including “selenium compound metabolism”, the “cell solute DNA sensing pathway”, “sulfur metabolism”, “starch and sucrose metabolism”, the “prolactin signaling pathway”, “phagosome”, “steroid biosynthesis”, and “arginine and proline metabolism” (Figure 2C). These pathways collectively contributed to mammary nutrient metabolism and cell renewal processes.

### 2.6. DEGs Associated with Late Lactation in the Liver and Mammary Glands from Dairy Goats Fed Diets with Varying Protein Levels

**Figure 3:**
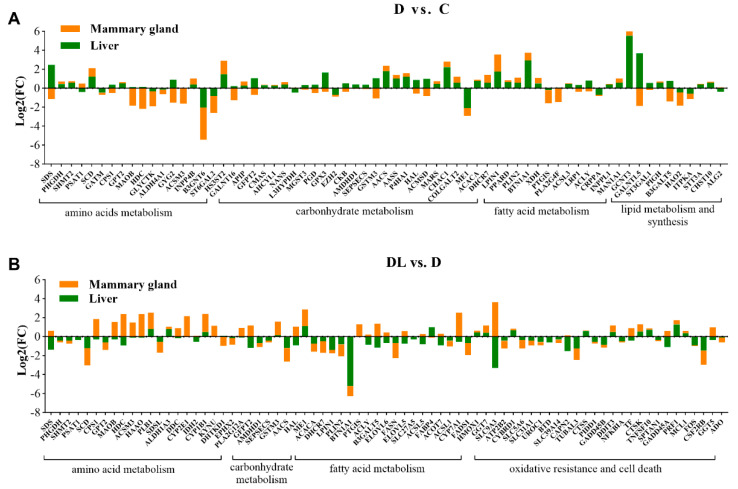
DEGs are involved in lactation processes in liver and mammary glands of dairy goats during late lactation. The effects of protein deficiency (**A**) and lysine supplementation (**B**) on differential gene profiles from both liver and mammary glands of dairy goats during late lactation. “C” represents the control group, namely protein-adequacy group; “D” represents the protein-deficient group; and “DL” represents the rumen-protected-lysine-supplemented protein-deficient group.

As is shown in Figure 3A, protein deficiency increased the expression of most amino-acid-metabolism-related genes in the liver, such as serine dehydratase (*SDS*), phosphoglycerate dehydrogenase (*PHGDH*), and serine hydroxymethyl-transferase 2 (*SHMT2*), but decreased the expression of monoamine oxidase B (*MAOB*), dehydrocholesterol reductase (*DHCR*), glycerate kinase (*GLYCTK*), Golgi associated, gamma adaptin ear containing, ARF binding protein 2 (*GGA2*, up-regulated), and receptor activity modifying protein 1 (RAMP1, up-regulated) in the mammary glands from the D vs. C groups. Apart from these, protein deficiency also elevated the expression of genes related to carbohydrate metabolism, such as adenosylhomocysteinase like 1 (*AHCYL1*) in the liver and solute carrier family 37 member 4 (*SLC37A4*) in the mammary glands from the D vs. C groups (Figure 3A). Also, protein deficiency was involved in lipid synthesis and secretion process in the liver, including *ACACA*, lipin (*LPIN*), perilipin 2 (*PLIN2*), *ACLY*, *PPARD*, and *XDH*, while mainly inhibiting the expression of *FABP7* and *SLC27A2* in the mammary gland tissue (Figure 3A).

In Figure 3B, the expression of numerous amino-acid-metabolism-related genes was down-regulated in the liver tissues from the DL vs. D (RPL supplementation vs. protein deficiency) groups, like *SDS*, *PHGDH*, *SHMT2*, guanidinoacetate N-methyltransferase (*GAMT*), and glutaminase 2 (*GLS2*). Likewise, in the mammary glands, RPL supplementation up-regulated the expression of several DEGs, including trehalase (*TREH*), glucokinase (*GCK*), glucose-6-phosphatase catalytic subunit 3 (*G6PC3*), and epoxide hydrolase 2 (*EPHX2*), compared to the protein-deficient group. However, these mammary genes in the DL vs. D groups that were involved in “arginine, proline, and phenylalanine metabolism” were down-regulated, which included amylo-alpha-1, 6-glucosidase, 4-alpha-glucanotransferase (*AGL*), phospholipase B1 (*PLB1*), phospholipase A2 group XIIA (*PLA2G12A*), and cytochrome P450 family 2 subfamily E member 1 (*CYP2E1*) (Figure 3B). As a central enzyme in carbohydrate metabolism, ME1 was up-regulated in both liver and mammary tissues (Figure 3B). Furthermore, the expression of genes related to fatty acid metabolism were reduced, including *ACACA*, *LPIN*, *PLIN2*, *ACLY*, fatty acid elongase 5 (*ELOVE5*), *ELOV6*, fatty acid synthase (*FASN*), and *FABP4* in the liver, while prostaglandin I2 synthase (*PTGIS*) was up-regulated in the mammary glands (Figure 3B). 

Furthermore, we validated some DEGs from mammary gland tissues that were closely related to milk production by real-time PCR. As is shown in Figure S1, the expression patterns of most selected mammary DEGs were consistent with the transcriptomic data with the exception of four DEGs, including (poly(ADP-ribose polymerase 1(*PARP1*), synaptosome associated protein 25 (*SNAP25*), transferrin receptor (*TFRC*), and transforming growth factor α (*TGFA*). Notably, in the context of protein deficiency, RPL supplementation highly rescued the expression of milk-production-related DEGs, including pyridoxal kinase (*PDXK*), suppressor of cytokine signaling 3 (*SOCS3*), and amphiregulin (*AREG*).

## 3. Discussion

Over the past century, the global livestock industry has experienced significant growth, accompanied by escalating environmental pressures. In China, carbon emissions from livestock have exhibited an annual increase from 2009 to 2016 [6]. It has been demonstrated that low-protein diets supplemented with single or mixed bypass AAs can effectively enhance ruminant feed utilization efficiency and mitigate environmental pollution while maintaining ruminant performance [4,7,8]. However, most related studies have primarily focused on early or mid-lactation, thus lacking a comprehensive understanding of the regulatory mechanisms governing milk production during the late-lactation period. Herein, we explored the effects of RPL supplementation on dairy goats at late lactation fed with low-protein diets and investigated the underlying molecular mechanism to advance our understanding of late-lactation regulation in dairy mammals.

In our study, it is a little unexpected that DMI showed little to no change among the three groups, suggesting the possible existence of alternative pathways for energy compensation within dairy goats at late lactation. However, N conversion was greater in goats fed RPL-supplemented diets than MP-deficient diets (DL vs. D groups) (17.94 vs. 14.43%), which is in line with the previous finding [9]. Aside from this, the decreased level of plasma NEFA under protein deficiency was rescued by RPL supplementation in dairy goats, suggesting supplementation with RPL might activate lipid metabolism to compensate for the energy requirement.

The liver plays a central role in energy metabolism. Here, our transcriptome study displayed remarkable changes in gene expression in the liver after lysine supplementation. For instance, the decreased expression of very-low-density lipoprotein receptor (*VLDLR*), caused by lysine sufficiency, was involved in extracellular VLDL uptake, and intracellular triglyceride (TG) storage, due to the positive relationship between *VLDLR* deficiency and lipid accumulation [10]. Also, RPL supplementation down-regulated *SCD* and fatty acid desaturase 1 (*FADS1*) in the liver, both of which participated in triglyceride and unsaturated fatty acid synthesis processes. This finding was aligned with a previous observation that both supplementation with methionine and lysine to low-protein diets reduced *SCD* and *FADS1* activities in the liver [7]. As a key enzyme within fatty acid synthesis, the down-regulation of acyl-CoA thioesterase 7 (*ACOT7*) would lead to a decline in hepatic long-chain-fatty-acid synthesis. Because fatty-acid-binding proteins (FABPs) play a crucial role in lipid transport between cellular organelles, the up-regulation of *FABP4* might activate lipolysis and suppress lipogenesis [11]. In addition, the down-regulation of acetyl-CoA carboxylase α (*ACACA*) (RPL supplementation vs. protein deficiency, namely the DL vs. D groups) might denote a lower rate of de novo TG synthesis. Thus, our findings provide a clue in that lysine availability contributes to maintaining ATP synthesis through active fatty acid metabolism. These down-regulated genes in the liver tissues from the RPL-supplementation vs. protein-deficient groups indicated that the supplementation with RPL mitigated the disturbances in hepatic lipid metabolism induced by protein deficiency, such as the “plasma uptake of fatty acid” and “de novo fatty acid synthesis”. In comparison to a normal diet, eight of the aforementioned 26 DEGs were significantly altered in late-lactating dairy goats fed with a low-protein diet. Among these, seven DEGs, including *PLIN2*, butyrophilin subfamily 1 member A1 (*BTN1A1*), *LPIN1*, *SCD*, (3-hydroxy-3-methylglutaryl-CoA reductase (*HMGCR*), pyridoxal phosphatase (*PLPP*), and a couple of *ACSL* family genes, were down-regulated, while the expression of *GPAT2* was elevated. GPAT2 plays a role in the production of lysophosphatidic acid, which is an essential step in the synthesis of triacylglycerol (TAG) and glycerophospholipid synthesis [12]. These findings suggested that inadequate protein intake stimulated active hepatic lipid synthesis and enhanced the synthesis and secretion of lipid droplets. An elevated level of liver AST may indicate a high-energy status but impaired liver function due to insufficient protein intake. However, RPL supplementation, in the context of inadequate protein intake, would possibly alleviate energy disturbances and maintain liver homeostasis through suppressing lipid biosynthesis. The elevated levels of circulating leptin and liver AST suggest a high-energy status and abnormal liver function, which might be caused by insufficient protein intake. However, due to inadequate protein intake, RPL supplementation alleviated energy disturbances and maintained liver homeostasis possibly by suppressing lipid biosynthesis.

Oxidative phosphorylation in cells is a most efficient mechanism for energy production, which provides a substantial amount of energy for cellular activities under normal physiological conditions [13]. In this context, protein deficiency activated a couple of cellular processes, such as the “inflammatory response”, “oxidoreductase activity”, and “oxidative–reduction processes”, while lysine availability inhibited oxidative stress. Importantly, two aldehyde dehydrogenase 1 family members (ALDH1A1 and ALDH1A3) are critical components of the antioxidant system, catalyzing the conversion of oxidized NAD(P)^+^ to reduced NAD(P)H [14]. The up-regulation of both enzymes in the liver of lysine-supplemented dairy goats suggests that lysine supplementation may mitigate the toxic effects of aldehydes on hepatic cells and further alleviate hepatic oxidative stress under these conditions. Furthermore, the liver can maintain relatively stable organ function within the body by synthesizing glucose through gluconeogenesis in the absence of protein intake [15]. This suggests that the mammary glands’ responses to nutrient intake levels are partially influenced by the hepatic self-regulation ability. This finding could, at least in part, explain why the transcriptional changes in mammary gland tissue were smaller than those of the liver in the same situation—protein deficiency.

AAs can act as nutritional signals that regulate milk lipid metabolism. For instance, supplementation with lysine, either alone or in combination with methionine, both increased the efficiency of milk production and the yield of milk fat in periparturient cows [16]. In our study, three DEGs (*PTGIS*, hematopoietic prostaglandin D synthase (*HPGDS*), and *PLA2G4F*) were overall decreased under protein deficiency, while lysine supplementation restored them to normal levels. Conversely, the up-regulation of *ACOX2* in response to lysine availability underscores its critical role in promoting fatty acid breakdown [17]. Furthermore, lysine activated several members of the phospholipase family, specifically *PLA2G12A*, *PLA2G15*, and phospholipase B1 (PLB1), in mammary gland tissues. Phospholipase A2 (PLA2) plays a limiting role in catalyzing the conversion of glycerophospholipid to generate arachidonic acid and is involved in hemolytic phospholipid biosynthesis [18]. Additionally, PLA2 participates in PPAR signaling pathways [19]. Therefore, lysine sufficiency may activate multiple processes in mammary lipid synthesis. This hypothesis is further supported by the up-regulation of mammary *FABP7* and solute carrier family 27 member 2 (*SLC27A2*). Overall, these findings suggested that protein deficiency influenced mammary lipid metabolism, and supplementation with lysine may promote the utilization of free fatty acids and triglyceride synthesis in the mammary gland by activating the PPAR signaling pathway, thereby regulating milk fat synthesis.

As is known, JAK–STAT signaling plays a crucial role in processes such as cell proliferation, apoptosis, and the immune response. It is noteworthy that no significant change related to the JAK–STAT signaling pathway was observed in the D group, but a big change was found in the RPL-supplementation group, compared to their respective controls, which was aligned with our previous finding [20]. In addition, the GSEA observed a significant enrichment in JAK–STAT signaling. Despite the lower *STAT5A/B* expression from lysine sufficiency in our transcriptomic data, it was unable to fully account for the activities of this JAK–STAT signaling. On the other hand, we found the up-regulation of the *SOCS3* gene, a component of the suppressor of cytokine signaling, that could be further activated by the JAK–STAT signaling pathway. However, in our study, the JAK–STAT signaling pathway was activated, possibly due to the varying abilities of the SOCS family proteins to affect the JAK/STAT signaling pathways [21,22]. Furthermore, as an upstream component of the JAK–STAT signaling pathway, the elevation of *GHR* suggested that lysine supplementation of a low-protein diet may activate the JAK–STAT signaling pathway through growth hormone. These findings supported the significant contribution of the JAK–STAT signaling pathway in maintaining late lactation when lactating goats were subjected to protein deficiency.

In this study, no common genes were found between the liver and mammary gland tissues, regardless of protein deficiency. Specifically, protein deficiency highly accelerated hepatic AA metabolism, such as the activation of Ser metabolism by up-regulated *SDS* and *PHGDH*, and the rapidly reversible transfer of one-carbon units from Ser to Gly by elevated *SHMT2* expression in the liver. Protein deficiency led to a decrease in the mammary expression of *MAOB* and histidine decarboxylase (*HDC*), reducing the breakdown of histidine. In this context, the up-regulation of *SLC37A4* promoted the transfer of glucose-6-phosphate from the cytoplasm to the endoplasmic reticulum, thereby facilitating glucose production. Since Ser, Gly, and His are gluconeogenic AAs, their distinct metabolic pathways in the liver and mammary glands might suggest that these AAs are cleared in the liver and subsequently utilized for gluconeogenesis or the regulation of blood sugar levels under protein deficiency. Upon RPL supplementation, the overall down-regulation of *SDS*, *SHMT2*, *PHGDH*, and *GATM* contributed to the inhibition of AA metabolism. In contrast, in the same context, the up-regulation of glucose-6-phosphatase catalytic enzyme (*G6PC*) and an AA transporter (*SLC5A10*) facilitated the transport of AAs outside the liver. Apart from these, numerous DEGs related to AA metabolism, such as tryptophan, arginine, proline, and alanine, were significantly enriched in mammary tissue from dairy goats at late lactation when fed RPL-supplemented diets. Based on the findings [23], lysine supplementation activated antioxidant metabolism-related pathways in mammary tissue, promoting the synthesis of antioxidant substances such as glutathione and taurine. This suggested a positive effect of lysine on promoting AA metabolism and defending against oxidative stress as well in mammary tissue.

## 4. Materials and Methods

### 4.1. Ethics Statement

All the experimental procedures and animal care were conducted in accordance with the guidelines of the Institutional Animal Care and Use Committee in Zhejiang University, Hangzhou, China (ZJU2017-0718).

### 4.2. Animals and Experimental Design

The experiment was conducted at Caiyang animal husbandry Co., Ltd., Lin’an, China. A total of thirty-three multiparous Guanzhong dairy goats (Bovidae), with an average weight of 60 ± 6.5 kg (mean ± SD) and a lactation period of 210 ± 10 days (mean ± SD), were used in this study. The goats were randomly divided into 3 blocks, each consisting of 11 goats, based on days in milk, milk yield, and parity. Fine-tuning was performed between blocks to ensure an equal number of pregnant goats across each treatment group. As is shown in Table S1, within each block, dairy goats were randomly assigned to one of the three groups fed with different diets: (1) the C group (control group, namely protein-adequacy group), fed with adequate metabolizable protein (MP), containing a slightly positive MP balance of 1.97 g of MP/day according to the National Research Council (NRC) (2007) [24]; (2) the D group (protein-deficient group), fed an MP-deficient diet, providing 88% of the MP in the above diet; and (3) the DL group (RPL-supplementation group), fed the MP-deficient diet and supplemented with 6 g RPL per goat daily. The experiment lasted for seven weeks, including a two-week adaptation period and a five-week experimental period. During the trial, the goats were fed once at 1300 h and milked once daily at 0630 h. Different levels of MP and AA digestible in the small intestine (AADI) within the different diets were obtained by varying the composition of the concentrate according to the NRC [24] (Table S1). RPL was quantitatively provided before feeding and wrapped with bean curd residue to avoid palatability issues.

### 4.3. Sample Collection and Measurements

Throughout the experiment, the total mixed ration and refusals were monitored and recorded daily. DMI was measured for two consecutive days biweekly. Samples of individual forage and concentrated feed ingredients were collected weekly and stored at −20 °C for subsequent analyses of dry matter, crude protein (CP), ether extract (EE), neutral detergent fiber (NDF), and acid detergent fiber (ADF) [25]. Blood samples were collected from the jugular vein before feeding on the sixth day of each week, centrifuged at 4 °C and 3500 rpm for 10 min to isolate the plasma. Milk samples from individual goats were collected, mixed, and weighed on the last two days of each week. Additionally, 4 out of 11 dairy goats from each group were randomly selected for liver-tissue and mammary-gland-tissue biopsy sampling. The tissue samples were quickly washed with normal saline, cut into small pieces, precooled in liquid nitrogen, placed into 2 mL centrifuge tubes, and then stored at −80 °C for further analyses of the mRNA abundances of genes related to milk synthesis.

### 4.4. Measurement of Milk Composition, Amino Acids, Plasma Metabolites, and Enzymes

In terms of milk samples, 40 mL milk was preserved with 0.6% potassium hexachloride (a commonly used milk preservative, D&F Control Systems, San Ramon, CA) and stored at 4 °C for further measurement. The milk measured here was collected from the last week of the experiment. The milk composition was analyzed using a 4-channel infrared spectrophotometer (Foss-4000; Foss Electric A/S, Hillerod, Denmark). The remaining milk and plasma samples were frozen at −80 °C for the measurement of free and hydrolyzed AAs using an AA analyzer (L-8900, HITACHI, Tokyo, Japan). Plasma glucose, cholesterol, NEFA (non-esterified fatty acids), and triglyceride concentrations were measured by an AutoAnalyzer 7020 instrument (HITACHI, Tokyo, Japan). The other plasma metabolites, including total protein, albumin (A), globulin (G), BUN, creatinine, and B-HB (β-hydroxybutyric acid), were analyzed by the corresponding colorimetric commercial kits purchased from Meikang Biotechnology Co., Ltd, Ningbo, China. The levels of three plasma enzymes, namely ALT, AST and ALP, and bilirubin were overall determined by the corresponding ELISA kits purchased from Meikang Biotechnology Co., Ltd, Ningbo, China.

### 4.5. Transcriptome Analysis

Liver and mammary gland tissues from four dairy goats per group were used for RNA sequencing (*n* = 4). A total of 3 mg RNA per sample, with an RNA integrity score higher than 8, was used as the input material for the RNA sequencing library construction. This was accomplished using the NEB-Next UltraTM RNA Library Prep Kit for Illumina (New England Biolabs, Ipswich, MA, USA), in accordance with the manufacturer’s instructions. After cluster generation, the library preparations were sequenced as paired-end (150 bp) reads at the Novogene Bioinformatics Institute on an Illumina HiSeq platform. The library quality was assessed using an Agilent 2100 BioAnalyzer (Agilent Technologies, Santa Clara, CA, USA). 

The protein-coding gene expression levels in each sample were estimated according to fragments per kilo-base of exon per million fragments, mapped and assessed with Cufflinks v2.1.1. DEG analysis among the three groups was performed using the DESeq2 R package. Transcripts with an FDR < 0.05 and FC > 2 were considered as DEGs. Gene ontology (GO) and Kyoto Encyclopedia of Genes and Genomes (KEGG) enrichment analysis for the DEGs were performed with the GOseq R package v1.44.0 and KOBAS software v 3.0, and a *P*-value < 0.05 was considered significant, using the Novomagic platform that is accessible at https://magic.novogene.com (accessed on 4 July, 2023).

### 4.6. RNA Extraction and Real-Time Quantitative PCR

Total RNA was extracted from mammary gland and liver tissues using the Trizol method (Aidlab, Beijing, China) for qPCR. The quality of the RNA was verified by agarose gel electrophoresis and its concentration was measured using a NanoDrop 2000 (Thermo Scientific, Wilmington, DE, USA). A total of 40 μL cDNA was synthesized using a reverse transcription kit (Cat. No. RR037A, Takara, Tokyo, Japan). In this study, β-actin was set as the reference gene. Fluorescence-based quantitative real-time PCR was performed using the TB GreenTM Premix Ex TaqTM kit (Cat No. RR420A, Takara, Tokyo, Japan). The PCR reaction was conducted on the Applied Biosystems^®^ 7500 Real-Time PCR Instrument (Applied Biosystems 7500, CA, USA) with a reaction volume of 20 μL. The relative mRNA abundance of genes was normalized to β-actin and calculated using the 2^−∆∆CT^ method. The sequences of the primers used in the PCR reactions are summarized in Table S5.

### 4.7. Statistical Analysis

The experimental data related to lactation performance, DMI, feed utilization efficiency, and N utilization efficiency were analyzed using the PROC MIXED model in SAS v9.2 software (SAS Institute, Cary, NC, USA). This analysis was conducted according to a complete random block design with repeated analysis of variance. Treatment, time, and the interaction between treatment and time were considered as fixed effects, whereas lactating goats were designated as random effects. The statistical model employed was as follows:

Yijk=μ+Bi+Tj+Wk+TWjk+Eijk

where *Yijk* is the free variable, *μ* = the overall mean, *Bi* = the random effect of block, *Tj* = the effect of treatment, *Wk* = the time effect, *TWjk* = the interaction between treatment and time, and *Eijk* = the random error. All results derived from the statistical analysis were expressed as least square means, and the significance of differences between means was evaluated using the Student–Newman–Keuls (S-N-K) test.

Furthermore, IBM SPSS Statistics (v20; IBM, Armonk, NY, USA) and GraphPad Prism (v9.0; GraphPad Software, San Diego, CA, USA) were utilized for analysis of gene expression data. One-way or two-way ANOVA was conducted, followed by Dunnett’s multiple comparisons test for the comparison of three or more groups.

## 5. Conclusions

**Figure 4:**
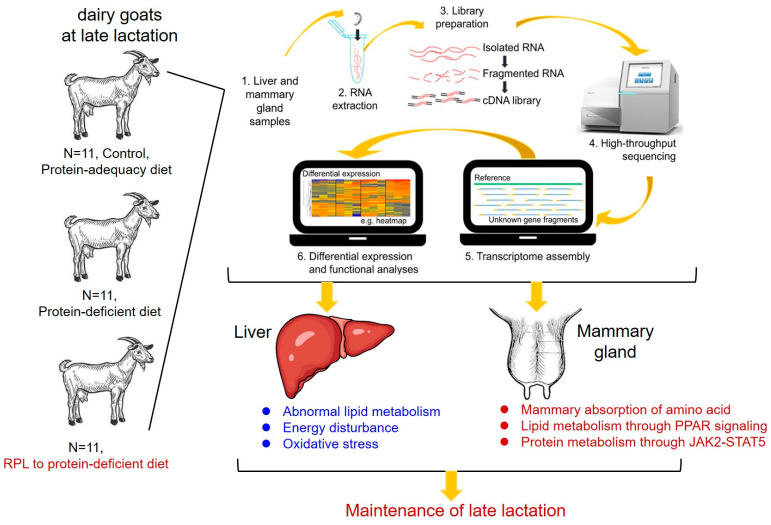
Schematic model depicting how RPL supplementation of a low-protein diet contributed to maintaining milk production and health at late lactation by integrated transcriptomics at cross-tissue levels. RPL: rumen-protected lysine.

In conclusion, our study indicates that RPL supplementation of a low-protein diet contributes to sustaining milk production and health during late lactation primarily through several interconnected mechanisms (Figure 4). Firstly, in terms of energy compensation, RPL reduced energy disturbances by inhibiting hepatic lipogenesis and promoted mammary lipid metabolism via activating the PPAR signaling pathway. More importantly, in the presence of protein deficiency, RPL played a crucial role in promoting milk protein synthesis within the mammary glands through elevating mammary protein metabolism and activating the protein-synthesis-related pathway, such as the JAK–STAT signaling pathway, etc. Thus, in the context of protein deficiency, these comprised events in mammary protein and lipid metabolism as well as the normal hepatic function from RPL supplementationoccurred, which provide some valuable insights into the molecular mechanism by which lysine supplementation facilitates the maintenance of milk production during late lactation in other dairy ruminants lacking protein.

## Figures and Tables

**Table 1:** Effects of RPL supplementation on lactation performance and N conversion in dairy goats.

Item	Treatment ^1^			SEM	*p*-Value		
	C	D	DL		T	W	T × W ^6^
DMI, kg/d	1.69	1.49	1.71	0.08	0.14	<0.01	0.09
Yield							
Milk, kg/d	0.76	0.60	0.69	0.10	0.52	<0.01	0.50
4% FCM ^2^, kg/d	0.89	0.79	0.76	0.08	0.54	<0.01	0.49
ECM ^3^, kg/d	0.99	0.87	0.85	0.09	0.51	<0.01	0.51
Milk protein, g/d	31.83	24.43	27.64	2.56	0.16	<0.01	0.41
Milk fat, g/d	36.99	38.63	31.30	4.06	0.58	<0.01	0.39
Lactose, g/d	39.25	32.23	27.32	4.38	0.24	<0.01	0.44
Milk composition, %							
Milk protein	3.68 ^a^	3.47 ^a^	4.01 ^b^	0.62	0.04	0.27	0.29
Milk fat	4.59	4.88	4.58	0.66	0.45	0.47	0.32
Lactose	4.67	4.45	4.47	0.67	0.10	0.32	0.32
Total solids	13.47	13.73	13.66	0.39	0.44	0.01	0.14
SCC, 10^3^/mL	7810	5653	5478	1416	0.40	0.11	0.12
MUN, mg/dL	23.55 ^a^	20.99 ^b^	21.77 ^b^	0.80	0.04	<0.01	0.99
FCR ^4^, %	49.71	41.70	38.86	0.08	0.94	0.24	0.92
N conversion ^5^, %	15.67	14.43	17.94	2.31	0.60	0.02	0.84

Different superscripts (a, b) within the same row indicate significant differences (*p* < 0.05). ^1^ “C” represents the control group, namely the protein-adequacy group; “D” represents the protein-deficient group; and “DL” represents the rumen-protected lysine (RPL)-supplemented protein-deficient group. ^2^ 4% FCM (fat corrected milk) (kg/d) = 0.4 × milk yield + 15 × fat yield. ^3^ ECM (energy corrected milk) (kg/d) = 0.3246 × milk yield + 13.86 × fat yield + 7.04 × protein yield. ^4^ FCR (feed conversion rate, feed efficiency) = milk yield/DMI. ^5^ N conversion = milk protein yield/CP intake. ^6^ T = treatment effect; W = week effect; and T × W = the interaction between treatment and week.

## Data Availability

The datasets produced and/or analyzed during the current study are available from the corresponding author on reasonable request.

## Supplementary Materials

The following supporting information can be downloaded at: https://www.mdpi.com/article/10.3390/ijms252111376/s1.
