# Supplementary material for: Integrated Transcriptomic Analyses of Liver and Mammary Gland Tissues Reveals the Regulatory Mechanism Underlying Dairy Goats at Late Lactation When Feeding Rumen-Protected Lysine
Source: Int J Mol Sci. 2024 Oct 23;25(21):11376. doi: 10.3390/ijms252111376 (PMC11546963; doi:10.3390/ijms252111376)
Supplement: Supplementary file 1 [file ijms-25-11376-s001.zip › ijms-3211268-supplementary.pdf]

International Journal of *Molecular Sciences*

MDPI

Supplementary Materials

Table S1. Ingredient compositions and nutrient levels of basal diets.

**Item**	**Diets**		
	C	D	DL
**Ingredient (% of dry matter)**			
Corn silage	21.65	21.65	21.65
Peanut vine	39.62	39.62	39.62
Bean curd residue	16.89	16.89	16.89
Maize	5.50	19.74	19.74
Soybean meal	5.54	0.23	0.23
Barely	5.58	1.68	1.68
Wheat	5.02	/	/
$CaHCO_4$	0.07	0.07	0.07
NaCl	0.07	0.07	0.07
Premix[1]	0.06	0.06	0.06
**Nutrients**			
ME, MJ/kg DM[2]	13.09	13.11	13.11
MP, g/kg DM[2]	120.97	106.54	106.54
CP, % of DM	11.09	10.31	10.31
NDF, % of DM	41.60	38.07	38.07
ADF, % of DM	21.28	19.29	19.29
Met (of AADI), g/d[3]	0.94	0.64	0.64
Lys (of AADI), g/d[3]	3.47	1.96	1.96
RPL/g	/	/	6

[1]premix contains (per kg of DM): 100 KIU VA, 250 KIU VD3, 2400 mg VE, 2000 mg nicotinic acid, 2000 mg Fe, 3000 mg Mn, 3000 mg Cu, 14000 mg Zn, 100 mg Se, 180 mg I, and 40 mg Co. [2]Values were calculated according to NRC (2007) models based on composition of the diets, DMI, milk yield and milk composition of goats during the trial. [3]Values were calculated based on composition of the diets and DMI data collected through adaptation period according to NRC (2007) models and tables containing the composition and nutritional value of feed materials. Met (AADI) and Lys (AADI) in the table only refers to the parts provided in the concentrate. “C” represents the control group, namely protein adequacy group; “D” represents the protein-deficient group; and “DL” represents the rumen-protected lysine supplementation to the protein-deficient group.

Table S2. Effects of RPL supplementation on free amino acid concentrations in blood from the jugular vein of dairy goats at late-lactation.

AA, mg/L	Treatment[1]			SEM	*P*-value
	C	D	DL		
EAA	73.75	66.15	63.70	3.41	0.49
Arg	12.74	11.50	11.74	1.49	0.37
His	4.96	5.03	6.58	0.53	0.43
Ile	6.53	6.39	5.17	0.45	0.40
Leu	8.07	8.01	6.41	0.49	0.28
Phe	4.90	4.68	4.75	0.22	0.93
Thr	18.43	19.28	18.84	0.79	0.92
Val	11.75	11.05	9.45	0.77	0.47
Lys	14.66	12.78	10.87	0.71	0.07
Met	2.11	1.97	1.71	0.51	0.52
NEAA	86.73	87.24	83.21	3.16	0.87
Ala	9.95	9.77	9.30	0.47	0.86
Asp	0.88	0.91	0.91	0.11	0.99
Cys	4.07	3.04	3.61	0.19	0.07
Glu	13.18	12.26	12.02	0.61	0.83
Glu	28.24	30.27	27.22	1.49	0.73
Pro	6.86	6.13	5.72	0.45	0.61
Ser	5.11	4.93	4.64	0.44	0.92
Tyr	7.27	6.30	6.48	0.41	0.62
**BCAA**	26.36	22.45	21.02	1.69	0.44
**TAA**	162.15	150.06	144.45	6.01	0.50

[1]"C" represents the control group, namely protein adequacy group; "D" represents the protein-deficient group; and "DL" represents the rumen-protected lysine supplementation to the protein-deficient group. [2]EAA = essential amino acids, NEAA = non-essential amino acids, BCAA = branched-chain amino acids (Val, Ile, Leu), TAA = total AAs. No significance was found in the above data.

Table S3. Effects of RPL supplementation on concentrations of hydrolyzed amino acids in milk from dairy goats at late-lactation.

AA, mg/L	Treatment[1]			SEM	*P*-value
	C	D	DL		
EAA	117.93	220.07	198.63	13.37	0.71
Arg	11.93[a]	14.95[b]	13.71[b]	1.11	<0.01
His	10.00[a]	12.57[b]	11.02[c]	0.67	<0.01
Ile	17.85	21.89	19.28	1.27	0.76
Leu	36.78[a]	46.24[b]	41.78[c]	2.66	<0.01
Phe	18.21	22.70	20.06	1.31	0.67
Thr	19.77	24.44	22.22	1.61	0.77
Val	24.51	30.83	27.26	1.87	0.67
Lys	9.98	11.91	9.94	2.17	0.77
Met	30.90[a]	36.93[b]	33.35[c]	0.73	<0.01
NEAA	200.04	245.50	222.15	14.22	0.71
Ala	13.17	15.64	14.97	1.13	0.82
Asp	28.39	33.66	31.26	2.25	0.87
Cys	2.93[a]	3.51[b]	3.77[b]	0.44	0.03
Glu	79.88[a]	97.40[b]	86.96[c]	5.16	<0.01
Gly	6.21[a]	8.38[b]	8.06[b]	0.68	<0.01
Pro	34.25[a]	43.67[b]	37.17[c]	1.04	<0.01
Ser	20.79	25.87	23.76	1.63	0.71
Tyr	14.41[a]	17.37[b]	16.20[b]	1.23	<0.01
BCAA	79.13[a]	98.96[b]	98.53[b]	4.77	<0.01
TAA	377.97	465.57	420.77	27.57	0.71

Different superscripts (a-c) within the same row indicate a significant difference ($P < 0.05$). [1]"C" represents the control group, namely protein adequacy group; "D" represents the protein-deficient group; and "DL" represents the rumen-protected lysine supplementation to the protein-deficient group. EAA = essential amino acids, NEAA = non-essential amino acids, BCAA = branched-chain amino acids (Val, Ile, Leu), TAA = total AAs.

Table S4. Effects of RPL supplementation on concentrations of physiological and biochemical items in plasma from dairy goats at late-lactation.

Items	Treatments [1]			SEM	P-value
	C	D	DL		
Protein metabolism					
Total protein, g/L	73.0	75.3	75.1	1.10	0.69
Albumin (A), g/L	31.2	30.0	31.2	0.58	0.68
Globulin (G), g/L	41.8	45.3	43.9	1.30	0.61
A/G	0.80	0.67	0.77	0.03	0.13
BUN, mmol/L	4.25[a]	3.85[b]	3.22[c]	0.09	0.04
Creatinine, μmol/L	52.0	53.1	52.1	1.74	0.97
B-HB, μmol/L	276	315	338	15.1	0.25
Energy substrates					
Glucose, mmol/L	3.68	3.52	3.56	0.09	0.78
NEFA, μmol/L	132[a]	110[b]	179[c]	4.9	< 0.01
Triglyceride, μmol/L	0.31	0.32	0.27	0.03	0.13
Cholesterol, mmol/L	2.99	2.57	2.67	0.11	0.33
Hepatic function					
ALT, U/L	18.7	17.4	16.7	0.75	0.59
AST, U/L	65.4[a]	81.4[b]	62.8[a]	2.81	0.02
ALP, U/L	146	206	149	33.2	0.74
Total bilirubin, μmol/L	0.86	0.78	0.81	0.09	0.94

Different superscripts (a-c) within the same row indicate a significant difference ($P < 0.05$). [1]"C" represents the control group, namely protein adequacy group; "D" represents the protein-deficient group; and "DL" represents the rumen-protected lysine supplementation to the protein-deficient group.

Table S5. RT-qPCR primers for validated genes in mammary gland tissues

Gene symbol	NCBI accession	Primer sequences（5◎3◎）	bp
AGL	XM_005678054.3	F: TTCTCTTCTCTGCCGCTTCTTTCAC<br>R: GACACACAACACTGCTCCTTCTCTC	92
ALDOC	XM_018064308.1	F: CAAGCGGGCTGAGGTGAATGG<br>R: GGCGTGGTTGGCAATGTAGAGG	103
AREG	XM_018049584.1	F: GGGACCACAGTGCTGACAGATTTG<br>R: AGGACAGTTCGCCACCAGGAG	87
CD44	XM_018059241.1	F: CGAATCCAGAAGCATCACCTCAGAC<br>R: GGTCCAGCGTTCTCCATAAGCAC	86
CDKN1A	XM_018039118.1	F: CTCGTGACTCGCTCCCCTGAG<br>R: CAGCCTGCGTTTGGAGTGGTAG	117
FBXO32	XM_005688865.3	F: CTGGTCCAAAGAGTCGGCAAGTC<br>R: AGGCAGGTCTGTGAAGGTGAGG	150
KAT2B	XM_018053238.1	F: CAACGACGACATCTCTGGCTACAAG<br>R: CAGCAGCGTCCTCCCAAACAC	124
LCN2	XM_018055847.1	F: TTCCAGGCCGACCAGTTCCAG<br>R: CCGTCTTCCTTCAGCTCGTAGTTG	122
MYBBP1A	XM_018064442.1	F: CGGATGCTGAAGACGCTGAAGG<br>R: ATGTCGCCCAGGAGGTCACAG	137
PARP1	XM_005690533.3	F: TCGCATATCAGCAAGTTACCCAAGG<br>R: AGGCACCTCCACACCATCCATAG	105
PDXK	XM_018051780.1	F: TCCTGACGGCTCTGTGGTGAC<br>R: TTGAGGTTGTTGGGGTGCTTGTG	132
QSOX1	XM_013970374.2	F: CGGCTGCTGATGCTGCTGTC<br>R: GCGGGTCGGAGGACGAGTAG	88
SGPL1	XM_005699154.3	F: TACTTCGGTGAGAGCGGCTACG<br>R: GCGATGACTGACAACTGAGGATTCC	128
SNAP25	XM_005687812.3	F: AGGTGAGCGGCATCATTGGAAAC<br>R: GTCGATCTGGCGGTTCTGTGTATC	83
SOCS3	XM_018063683.1	F: GACTGCGTGCTCAAGCTGGTG<br>R: GGGCGAGGAGGAGGGTTCAG	90
STAT5A	XM_018065112.1	F: GCGGAAGCAGCAGACCATCATC<br>R: GCCAACTTCTCACACCAGGACTG	129
TCF7L2	XM_018041220.1	F: CACCTGGCACCGTAGGACAAATC<br>R: AACCTGGACATGGAAGCGTTGAC	142
TFRC	XM_013963470.2	F: CCACCATCTCAGTCATCAGGATTGC<br>R: AGAGTCTGTTCCCCAAGTGCTAGG	120
TGFA	XM_013967513.2	F: CCTGGCTGTCCTCATCATCACATG<br>R: GTGGCAGCAAGCAGTCCTTCC	136
ACTB	XM_018039831.1	F: TGCGGCATTCACGAAACTAC<br>R: TGTTGGCGTAAAGGTCCTTG	76

AGL: amylo-α-1, 6-glucosidase; *ALDOC*: aldolase; *AREG*: amphiregulin; *CD44*, CD44 molecule; *CDKN1A*: cyclin dependent kinase inhibitor 1A; *FBXO32*, F-Box protein 32; KAT2B, lysine acetyltransferase 2B; *LCN2*, lipocalin 2; *MYBBP1A*, MYB binding protein 1a; *PARP1*, poly(ADP-ribose) polymerase 1; *PDXK*, pyridoxal kinase; *QSOX1*, quiescin sulfhydryl oxidase 1; *SGPL1*, sphingosine-1-phosphate lyase 1; *SNAP25*, synaptosome associated protein 25; *SOCS3*, suppressor of cytokine signaling 3; *STAT5A*, signal transducer and activator of transcription 5A; *TCF7L2*, transcription factor 7 like 2; *TFRC*, transferrin receptor; *TGFA*,transforming growth factor α; *ACTB*, β–actin.

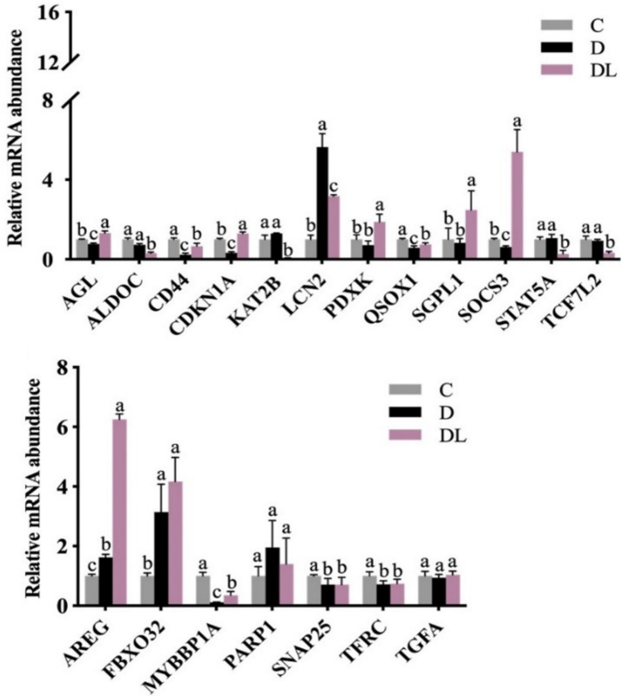

Figure S1 The DEGs mostly related to milk production from mammary glands were validated by real-time PCR. *AGL*, amylo-alpha-1, 6-glucosidase; *ALDOC*, aldolase; *CD44*, CD44 molecule; *CDKN1A*, cyclin dependent kinase inhibitor 1A; *KAT2B*, lysine acetyltransferase 2B; *LCN2*, lipocalin 2; *PDXK*, pyridoxal kinase; *QSOX1*, quiescin sulfhydryl oxidase 1; *SGPL1*, sphingosine-1-phosphatelyase 1; *SOCS3*, suppressor of cytokine signaling 3; *STAT5A*,signal transducer and activator of transcription 5A; *TCF7L2*, transcription factor 7 like 2; *AREG*, amphiregulin; *FBXO32*, F-box protein 32; *MYBBP1A*, MYB binding protein 1a; *PARP1*, poly(ADP-ribose) polymerase 1; *SNAP25*, synaptosome associated protein 25; *TFRC*, transferrin receptor; *TGFA*, transforming growth factor α. Different superscripts (a-c) within the same row indicate a significant difference ($P < 0.05$). "C" represents the control group, namely protein adequacy group; "D" represents the protein-deficient group; and "DL" represents the rumen-protected lysine supplementation to the protein-deficient group.
